# Valorization of porcine by-products: a combined process for protein hydrolysates and hydroxyapatite production

**DOI:** 10.1186/s40643-022-00522-6

**Published:** 2022-03-21

**Authors:** Sandra Borges, Clara Piccirillo, Francesca Scalera, Rui Martins, Ana Rosa, José António Couto, André Almeida, Manuela Pintado

**Affiliations:** 1 Universidade Católica Portuguesa, CBQF - Centro de Biotecnologia e Química Fina – Laboratório Associado, Escola Superior de Biotecnologia, Rua Diogo Botelho 1327, 4169-005 Porto, Portugal; 2 Institute of Nanotechnology/NANOTEC, National Research Council, Lecce, Italy; 3 ETSA, Empresa Transformadora de Subprodutos, Loures, Portugal

**Keywords:** Porcine by-products, Bioactive peptides, Enzymatic hydrolysis, Natural hydroxyapatite, Nanomaterial

## Abstract

The meat industry generates large amounts of by-products that are costly to be treated and discarded ecologically; moreover, they could be used to extract high added-value compounds. In this work, we present an innovative combined process which allowed the parallel extraction of both organic and mineral compounds; more specifically protein hydrolysates and single-phase hydroxyapatite were obtained. The protein hydrolysates, extracted through an enzymatic hydrolysis with alcalase, showed a degree of hydrolysis of 53.3 ± 5.1%; moreover, they had a high protein content with peptides with molecular weight lower than 1.2 kDa. Their antioxidant activities, measured with ABTS and ORAC tests, were 21.1 ± 0.5 mg ascorbic acid equivalent/g of dry extract and 87.7 ± 6.3 mg Trolox equivalent/g of dry extract, respectively. Single-phase hydroxyapatite, obtained with a simple calcination at 700 °C on the residues of the hydrolysis process, showed a Ca/P ratio close to the stoichiometric one (1.65 vs. 1.67) and presented a nanometric structure. This study reports a simple and feasible process for the valorization of porcine by-products in a large-scale up generating products with potential applications for environment remediation, biomedicine, nutrition and catalysis/bioenergy.

**Graphic Abstract:**

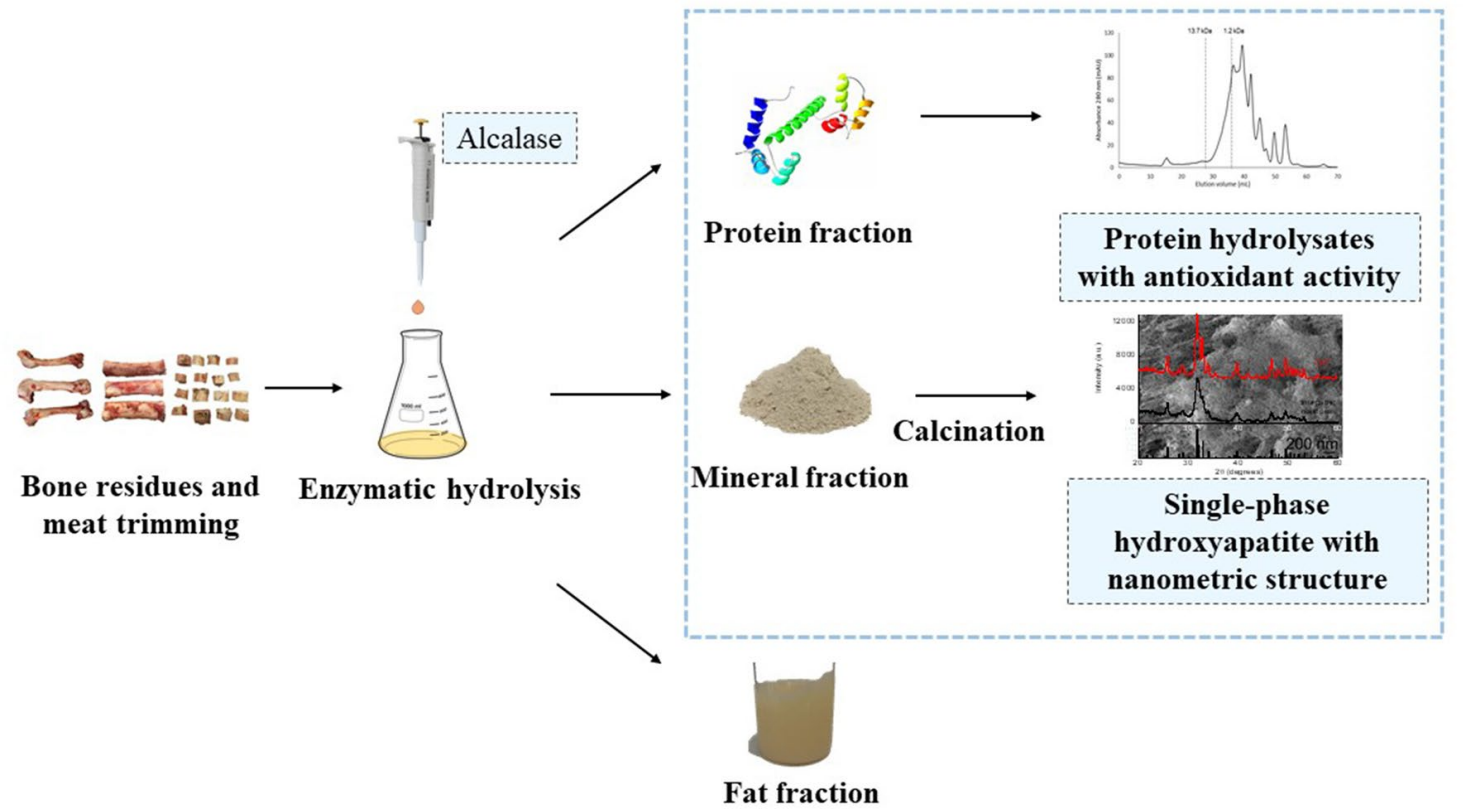

## Introduction

The agri-food industry generates massive quantities of by-products, which can be an environmental issue and should be properly addressed. By-products from slaughter and processing of pigs represent approximately 44% of the total live weight of the animal. These by-products are commonly used as animal feed, fertilizers and also in the production of biogas; these applications, however, have a relatively low economic value (Lapeña et al. 2018). Nevertheless, this residual raw material has a high nutritional value containing large amounts of protein, lipids and minerals, which have potential to generate high value-added ingredients. Therefore, a better exploitation of these meat by-products is crucial for sustainability and the circular economy. Such valorization, as an alternative to a simple reuse of the by-products, could also provide novel ingredients and products that innovate the food industry (Fu et al. 2018).

Several studies show that meat residues such as trimmings and bones contain high quantities of proteins, particularly collagen (Toldrá et al. 2016), whose potential is well known; indeed, collagen, is used in various fields, including biomedicine and cosmetics (Ferraro et al. 2017). In addition to this, collagen can also be used as a source of smaller bioactive molecules, which can be obtained with a process of hydrolysis (Ahmed et al. 2020), as proteins are broken down into smaller and more water-soluble peptides and free amino acids. With the hydrolysis, there is an increase in protein recovery; moreover, valuable compounds such as protein hydrolysates are produced.

Protein hydrolysis can be a suitable method to extract proteins from meat residues; the process can be made more efficient if performed with appropriate enzymes (Toldrá et al. 2016). Enzymatic hydrolysis can be performed using endogenous enzymes (digestive enzymes) or exogenous enzymes (commercially available) (Aspevik et al. 2017). The process, however, is more specific and reproducible with exogenous enzymes; hence, this represents a good option to produce food-grade and well-defined protein hydrolysates (PH). Despite the additional costs of commercial enzymes, the process is still economically viable, since the products have potential to achieve higher-paying markets compared for example with products based on rendering (Aspevik et al. 2017).

Enzymatic hydrolysis has been used to obtain antioxidant compounds from various animal by-products including duck (Li et al. 2020), goat (de Queiroz et al. 2017) and bovine (Zou et al. 2019). Previous studies also showed that porcine peptides could be an antioxidant source, namely peptides from porcine hemoglobin (Chang et al. 2007; Álvarez et al. 2012), skin (Li et al. 2007), myofibrillar protein (Saiga et al. 2003) and other porcine tissues (colon, appendix, rectum, pancreas, heart, liver, and lung) (Damgaard et al. 2014). These bioactive peptides derived from pork by-products with potential health-promoting effects have a wide range of promising applications, such as nutraceuticals for pets and humans, as well as in cosmetic and pharmaceutical formulations (Aspevik et al. 2017).

Animal by-products, besides being a valuable protein source, can also be an important basis to extract calcium phosphates (CaP), particularly hydroxyapatite (HAp). HAp, whose formula is Ca_10_(PO_4_)_6_(OH)_2_, is the major inorganic component of hard tissues (Lü et al. 2007); more specifically, in animal bones its content is over 60%. HAp is widely used in the biomedical area for bone regeneration due to its excellent properties such as biocompatibility, bioactivity, osteoconductivity and also noninflammatory and nonimmunogenicity behaviors (Barakat et al. 2009). In addition to this, HAp has other applications; in fact, it can also be used for environment remediation, as it can remove bivalent heavy metals from contaminated wastewaters and soils (Khan et al. 2020; Nie et al. 2020; Safavi et al. 2020). The synthetic HAp involves a chemical reaction between calcium and phosphorus in appropriate conditions; this approach, however, is not sustainable in the long term, due to the increasing demand of phosphorus for agriculture (Santos et al. 2019). It is therefore important to consider innovative and sustainable sources of HAp; indeed HAp extraction from food by-products has been explored, for instance from bovine bones (Barakat et al. 2009) and porcine bones and teeth (Lü et al. 2007). In other cases, for instance from fish bones (Piccirillo et al. 2013) a mixture of HAp and other CaP compounds was obtained; this was because the ratio between Ca and P in the bones was smaller than the stoichiometric one (1.67). Literature data showed that natural HAp and/or CaP are suitable for biomedical applications (i.e., bone substitutes, grafting, etc.). Currently, some bone substitutes of animal origin are commercially available, as is the case of Apatos® which is derived from a cortical porcine bone in the form of particles.

As mentioned above, processes to recover/extract protein hydrolysates or CaP have been considered; literature, however, does not report on a combined process to extract both compounds from meat by-products. Such a process would be important to have a more complete valorization of these by-products.

This work explores for the first time a combined process for the valorization of porcine by-products (bone residues and meat trimmings), which includes a bioprocess (enzymatic hydrolysis) followed by a thermal treatment. This approach allows the simultaneous extraction of organic and mineral fraction as added-value compounds (PH and CaP).

The obtained products (PH and CaP) were characterized by several analytical techniques, to evaluate their composition and to explore their potential to food/feed, medical and environmental applications. This work shows it is possible to perform a simultaneous extraction of several high added-value compounds from the same by-products of pigs slaughter and processing, usually discarded.

## Materials and methods

### Materials and reagents

Porcine by-products (meat and bones) were obtained by ETSA (Loures, Portugal), a company specialized in the collection of animal by-products from conventional centers including slaughterhouses. All reagents were purchased from Sigma-Aldrich (USA) unless mentioned otherwise.

### Combined process of protein and hydroxyapatite extraction from porcine by-products

**Fig. 1:**
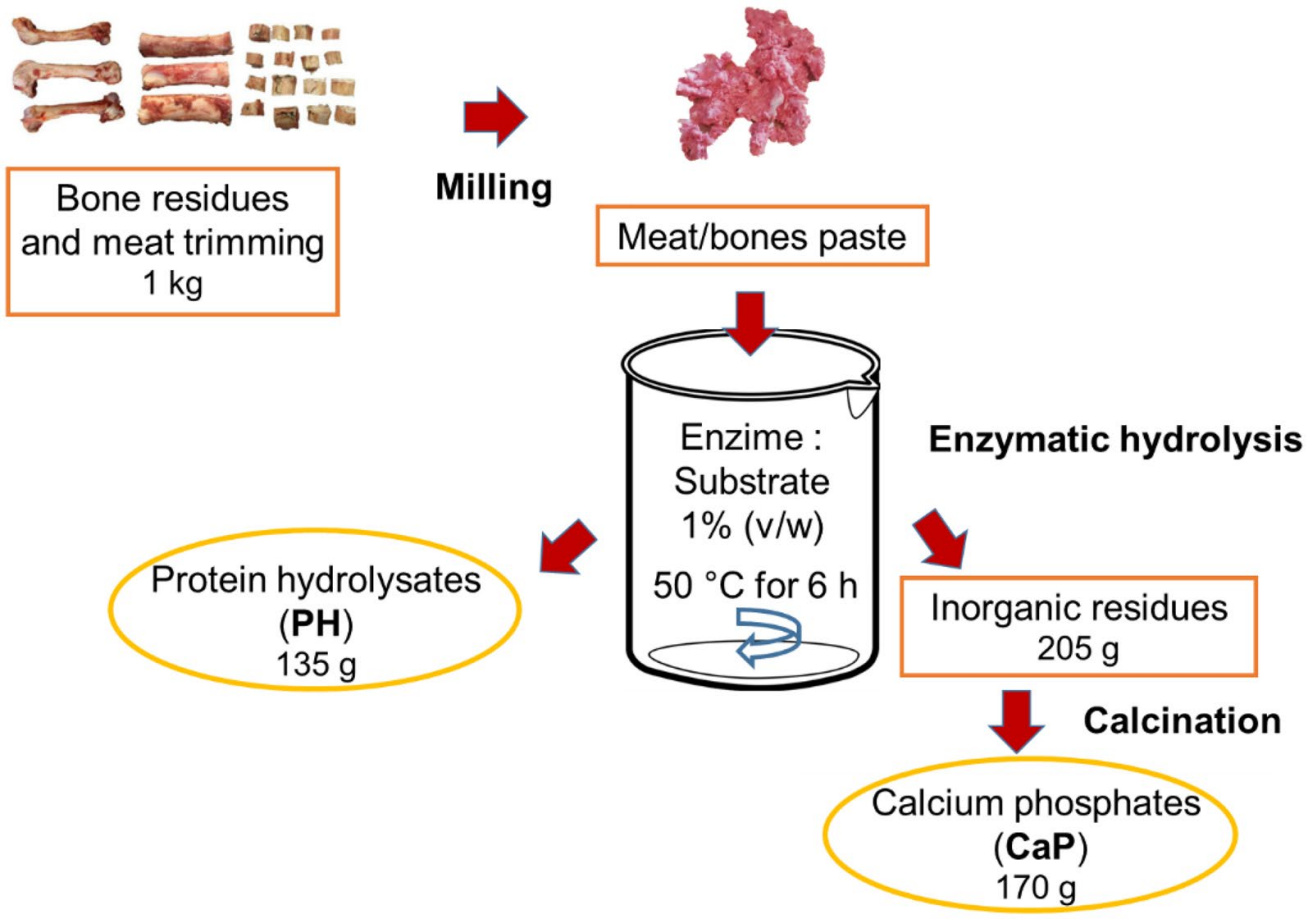
Scheme of the combined process to extract proteins and CaP

A scheme of the process employed to extract proteins and CaP is shown in Fig. 1.

A mixture of porcine bones and meat trimmings were used; they were ground at room temperature, to obtain a pulp-like meat paste, which was then submitted to hydrolysis in order to extract the PH and CaP.

To extract the proteins by hydrolysis, water was added to the meat/bone paste in a ratio of 1:1. Prior to the enzymatic hydrolysis, the pH was adjusted to 8.0 with 1 M NaOH. The substrate was hydrolyzed by alcalase at a ratio of enzyme:substrate of 1% (v/w) at 50 °C for 6 h. During the hydrolysis, the pH of the reaction mixture was kept constant by continuous addition of NaOH. Enzymatic reaction occurred at the optimal pH and temperature conditions described for alcalase (Borrajo et al. 2020b; Sousa et al. 2020). The mixture was then submitted to centrifugation (5000*g*, 5 min) (Gyrozen 1248, Korea) in order to separate and obtain three phases: the fat in the upper phase, the intermediate water phase containing the soluble protein, and the lower phase containing the mineral part. The upper phase containing the fat was discarded and the protein fraction was collected and stored at −20 °C for further analysis. The mineral fraction or inorganic fraction was washed in water, dried at 120 °C in an oven, and ground in a coffee mill, obtaining a white bone powder. Then, to remove the residual organic fraction and obtain pure minerals, the powder was calcined at 700 °C. The heating ramp was 5 °C/min and the annealing time was 1 h (Piccirillo et al. 2013).

### Characterization of porcine protein hydrolysates

#### Determination of degree of hydrolysis

The hydrolysis efficiency was determined through the degree of hydrolysis (DH), which was assessed by measuring the free amino groups by reaction of 2,4,6-trinitrobenzenesulfonic acid solution (TNBS) (Sousa et al. 2020). Briefly, a reaction mixture with 50 μL of PH extract, 125 μL of 200 mM sodium phosphate buffer (pH 8.2) and 50 μL of TNBS at 0.025% were placed in a 96-well microplate (Sarstedt, Germany). The microplate was incubated at 45 °C for 1 h and the absorbance was measured at 340 nm using a Multiskan GO plate reader (Thermo Scientific, USA). L-leucine (0.078–2.5 mM) was used to generate a standard curve. Three replicates were recorded. The DH was determined by following formula:

$$DH \left(\%\right)=100*\frac{{L}_{t}-{L}_{0}}{{L}_{max}-{L}_{0}},$$

where *L*_*t*_ is the amount of amino groups released after a hydrolysis time equal to t, *L*_*0*_ is the amount of amino groups in the sample at initial hydrolysis time (blank) and *L*_*max*_ is the maximum amount amino groups existing in porcine by-products. The *L*_*max*_ was obtained by acid hydrolysis of porcine by-products with 6 M HCl at 105 ºC for 24 h. Then, the acid-hydrolyzed sample was filtered and the supernatant was neutralized with 6 M NaOH before amino group acids assessment.

#### Composition analysis

The composition analysis was performed according to the Association of Official Analytical Chemists procedures (AOAC 1995). The moisture was determined at 105 °C for 24 h. The ash content was determined at 550 °C for 5 h. The protein content was measured using the Kjeldahl method and the nitrogen to protein conversion factor used was 6.25. The protein content was expressed on a dry weight basis. All measurements were performed in triplicate.

#### Molecular weight distribution

The molecular weight (MW) distribution of porcine PH extract was determined by a fast protein liquid chromatography (FPLC) (Sousa et al. 2020). An aliquot (100 µL) of filtered samples was injected in a AKTA pure 25 L system, from GE Healthcare Life Sciences (Freiburg, Germany), coupled with two gel filtration columns: Superdex 200 increase10/300 GL and Superdex peptide, 10/300 GL. The eluent used was 0.025 M phosphate buffer (pH 7.0), 0.15 M sodium chloride and 0.2 g/L of sodium azide. The flow rate was 0.5 mL/ min and elution was monitored at 280 nm. A MW standard curve was established using thyroglobulin (669 kDa), ferritin (440 kDa), aldolase (158 kDa), conalbumin (75 kDa), ovalbumin (44 kDa), carbonic anhydrase (29 kDa), ribonuclease A (13.7 kDa) and a whey peptide (1.2 kDa). The analysis was performed in duplicate and the results were expressed in milli Absorbance Units (mAU) per eluted volume (mL). The software used to evaluate the results was UNICORN 7.0.

#### Analysis of antioxidant activity

##### ABTS scavenging assay

The ability of free radical-scavenging by porcine PH extract was evaluated through 2,2-azino-bis-3-ethylbenzothiazoline-6-sulphonic acid (ABTS) radical decolourization assay (Re et al. 1999). The radical cation was formed by reacting ABTS with potassium persulfate. Then, 1 mL of ABTS solution was reacted with the sample for 6 min and then the absorbance was measure at 734 nm. A calibration curve was prepared with ascorbic acid in the range of 0.063–0.250 mg/mL and all the determinations performed in triplicate. Results were expressed as mg ascorbic acid equivalent/g of dry extract.

##### ORAC assay

The measurement of oxygen radical absorbance capacity (ORAC-FL) was performed (Ou et al. 2001). The porcine PH sample were dissolved in 75 mM phosphate buffer (pH 7.4) and the solution was placed in a black 96-well microplate (Nunc, Denmark), mixed with 120 μL of fluorescein (70 nM) and incubated at 40 °C for 10 min. Then, 60 μL of 2,2’-azobis(2-amidinopropane) dihydrochloride (AAPH) solution (14 mM) was added to the mixture, and the fluorescence was recorded using a microplate reader (Synergy H1, USA) at excitation and emission wavelengths of 485 and 528 nm, respectively, for 140 min at intervals of 1 min. The area under curve (AUC) was calculated for each sample by integrating the relative fluorescence curve. Trolox (9.98 × 10^−4^–7.99 × 10^−3^ μmol/mL) was used as the standard and regression equations for Trolox and samples were calculated. The ORAC values were determined by the ratio of sample slope to the trolox slope obtained in the same assay. Final ORAC values were expressed as mg Trolox equivalent/g of dry extract.

### Characterization of the inorganic fraction—CaP

The inorganic fraction, separated and calcined as described above, was characterized with the following techniques.

#### Thermal analysis (TGA)

The thermogravimetric analysis of the inorganic residues (prior to calcination) was performed using SDT Q600 (TA Instruments) TGA equipment, with an air flow rate of 100 ml/min and a heating ramp of 5 °C/min.

#### Determination of Ca and P

Powders were dissolved in HNO_3_ (Merck, Germany) to determine calcium and phosphorus concentrations.

Calcium content was measured by flame atomic absorption spectrometry (Solaar 969 AA Spectrometer, Unicam, UK). A La solution (Spectrosol, England; 4 g/L) was added to the samples acid solution to prevent ionization interference. A calibration curve of Ca (0.5–2.0 mg/mL) was prepared by dilution of the respective atomic absorption standard solution (Spectrosol, England). Phosphorus concentration was measured by a spectrophotometric method, using a Spectroquant phosphorus reagent kit (Merck, Germany). A calibration curve of standard K_2_HPO_4_ was used and all measurements were performed at 400 nm. The assays were performed in duplicate. The results were expressed in % (g of Ca or P/100 g of sample); Ca/P molar ratio was also calculated.

#### Phase analysis

Phase analysis of the inorganic residues and of the calcined powder was determined by X-ray diffraction (XRD). A X’Pert PRO MRD diffractometer was used, with CuKα radiation; the diffraction patterns were acquired with a step size of 0.005° and a count time of 100 s; an interval between 20 and 60° was considered. The registered patterns were compared with the JCPDF standard file 01-072-1234 for HAp.

Samples were also analyzed by Fourier transformed infrared spectroscopy (FTIR) in a spectrum series Perkin Elmer spectrometer (ABB, Switzerland) equipped with an attenuated total reflectance (ATR) sampling accessory (PIKE technologies, USA) and a diamond/ZnSe crystal. All spectra were acquired between 500 and 4000 cm^−1^.

#### Sample morphology

The morphology of the samples was analyzed with the scanning electron microscopy (SEM) technique, using a Carl Zeiss Merlin instrument, equipped with a Gemini II column and an integrated high efficiency In-lens for secondary electrons. Before the analysis, the samples were sputtered with gold to prevent charge accumulation.

## Results and discussion

### Yield of the process

Figure 1 shows the scheme of the process, as well as the yield for each step. It can be seen that, starting from 1 kg of material, the amount of PH extracts is about 135 g–13.5%; the inorganic residues, on the other hand, are about 205 g–20.5%.

### Porcine protein hydrolysates

#### Characterization of porcine PH

Proteins of animal origin are known for their nutritional properties as a crucial source of amino acids; in fact, these are released upon digestion or industrial processing from the parent protein. Meat is one of the most studied sources for the production of bioactive peptides due to the presence of high-quality proteins (Albenzio et al. 2017). Some industrial food-grade proteinases, namely alcalase, flavourzyme, bromelain and papain, have been used for the generation of hydrolysates of porcine proteins (Chang et al. 2007; Liu et al. 2010; Wang et al. 2008; López-Pedrouso et al. 2020).

Alcalase is very noteworthy from an industrial standpoint, because of its activity/stability at alkaline pH values, having a wide application. Alcalase has been used as additive in detergent formulations, it can be employed in meat tenderizing, dehairing and bating leather, cheese flavor improvement, baked manufacture, or enhancing digestibility of animal feeds. The reaction of protein hydrolysis catalyzed by alcalase has a strong tendency to develop a hydrolysate with many peptides of small size, due to the extensive range of amino acids that this enzyme can recognize. Therefore, the broad enzyme selectivity and specificity allows the use of alcalase in a variety of protein substrates, yielding a high protein hydrolysis degree (Tacias-Pascacio et al. 2020). Moreover, there is growing evidence that alcalase on its own shows a higher ability for hydrolysis in comparison with other commercial enzymes. As demonstrated in the work of Ahmadifard et al. (2016), the enzymatic hydrolysis of rice bran protein concentrate and soybean protein showed that alcalase presented a higher capability for hydrolysis of approximately 10 times higher than other enzymes.

Based on these data, the hydrolysis of porcine by-products was accomplished by alcalase, a serine endopeptidase from *Bacillus licheniformis*.

A complete characterization of the PH extract was performed; the results are reported in Table 1.

**Table 1:** Composition of the PH extracts

	DH (%)	Dry matter (% w/w)	Protein content (% w/w dry basis)	Ashes (% w/w dry basis)	Other components (% w/w dry basis)	Yield of the process (%)
PH extracts	53.1 ± 5.1	10.3 ± 0.0	70.4 ± 2.4	13.9 ± 0.4	5.4	13.5

The DH is an indicator widely used to compare hydrolysis efficiency among different protein hydrolysates. The DH of PH extracts from these food by-products was 53.3 ± 5.1%. This value is higher than achieved for other pork tissue hydrolysates also produced by alcalase. Liu and colleagues (Liu et al. 2010) hydrolyzed porcine plasma protein with 2% (w/w) alcalase for 5 h, showing a DH of 17.6%. Chang and collaborators (Chang et al. 2007) performed the proteolytic reaction of porcine hemoglobin with 2.0% alcalase, and after 6 h obtained hydrolysates with a DH less than 10%. Verma and collaborators (Verma et al. 2017) carried out the proteolytic reaction of porcine liver with 1% (w/w) alcalase over 6 h, and the hydrolysates had a DH of 23.56%.

Regarding the composition, the dry matter of PH extracts was 10.3 ± 0.0%; this is in agreement with literature, which reports that the dry matter content in the porcine hydrolysates can vary between 5.9 and 13.8%, depending on pork tissue types used for hydrolysis (Damgaard et al. 2014). As expected, this fraction is protein-rich, showing a content of 70.4 ± 2.4% (w/w dry basis), which is within the values described for porcine hydrolysates; for instance, hydrolyzed swine mucus protein has approximately 59% crude protein and hydrolyzed swine liver has ca. 78% crude protein (dos Santos Cardoso et al. 2020). The enzymatic hydrolysis is able to produce peptides which are more water-soluble than the intact proteins, so it was possible to obtain a high protein recovery. PH extracts showed an ash content of 13.9 ± 0.4% (w/w dry basis), which indicates a large amount of minerals. Minerals are essential for human and animal health because they are important for several functionalities, such as building strong bones, imparting nerve impulses, producing different hormones and also regulating the heartbeat (Gharibzahedi et al. 2017). The proportion of the remaining components was calculated by difference; it is likely that some lipids are present in the extracts.

Considering these results, this porcine-derived PH extract showed a high nutritive quality.

#### Peptide profile analysis

**Fig. 2:**
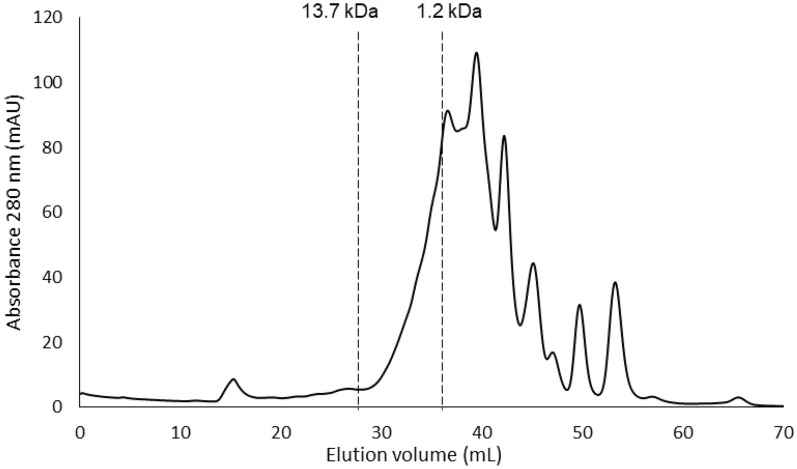
Size-exclusion FPLC profile of porcine protein hydrolysates obtained upon hydrolyzing with alcalase. Molecular weight markers of 13.7 kDa and 1.2 kDa are indicated

Besides DH measurement, an extremely important parameter used for characterization of PH extracts is the molecular weight distribution of the peptides. This is a direct analysis of the peptides and protein content, unlike the DH which is a measure relative to the raw material. Enzymatic hydrolysis decreases the molecular weight of intrinsic protein and increases the number of ionizable groups, resulting in novel peptides. Porcine PH extracts were analyzed by gel filtration chromatography to determine the peptide length distribution (Fig. 2). The chromatogram revealed that alcalase produced hydrolysates with small peptides. According to the calibration curve of standards, PH contained peptides with molecular weight lower than 13.7 kDa, with a high contribution of peptides with molecular weight smaller than 1.2 kDa. This result confirmed that proteins in porcine by-products were degraded by alcalase into low MW peptides or free amino acids. Fu and co-authors (Fu et al. 2019) also showed that porcine hemoglobin and whole blood treated with different proteases, such as alcalase, generated peptide fractions with low MW, most of them below 1 kDa.

This is expected to be beneficial for the antioxidant activity, since small peptides have been revealed to have higher activity than peptides with high MW (Ajibola et al. 2011; Irshad et al. 2015).

#### Antioxidant activity of porcine protein hydrolysates

The search for bioactive protein hydrolysates from meat by-products has been instigated by the growing interest in the development of functional foods, along with the control of food lipid oxidation. These valuable compounds could revalue the by-products, while mitigating the environmental and economic issues caused by the meat industry. Furthermore, the antioxidant properties related with these compounds offers an alternative to synthetic additives, which are linked with adverse effects on human health (Borrajo et al. 2020a).

Some animal proteins have been used as substrates for alcalase hydrolysis, such as sheep visceral protein, which produced a PH with an antioxidant activity of 68% (Meshginfar et al. 2014). In vivo and in vitro antioxidant capacity of porcine splenic hydrolysate produced using alcalase, suggested that porcine splenic hydrolysates improve the antioxidant status in rats by increasing hepatic catalase and glutathione peroxidase activities (Han et al. 2014).

Thus, the antioxidant capacity of porcine protein hydrolysates prepared with alcalase was evaluated by ABTS and ORAC assays; results are reported in Table 2.

**Table 2:** Antioxidant activity of the protein hydrolysates

	ABTS (mg ascorbic acid equivalent/g dry extract)	ORAC (mg Trolox equivalent/g dry extract)
PH extracts	21.1 ± 0.5	87.7 ± 6.3

The ABTS method evaluates the ability of an antioxidant compound to transfer electrons or donate hydrogen atoms to a preformed ABTS radical cation, whose change of color causes a decrease in absorbance (Re et al. 1999). The value of the radical scavenging activity of porcine PH extract was 21.1 ± 0.5 mg ascorbic acid equivalent/g of dry extract (55.16 ± 1.34 in terms of % radical scavenging activity—%RSA). This antioxidant activity is in agreement with the values observed for other porcine hydrolysates extracted with alcalase. Porcine liver protein hydrolysates showed a RSA ranged from 38.43 to 74.62% for 0–6 h reaction time (Verma et al. 2017). Damgaard et al. (2015) tested the antioxidant capacity of different porcine tissue hydrolysates (heart, colon and neck) using a mixture of alcalase and protamex; they registered values of RSA between 37.9 and 49.6%.

The activity of hydrolysates to scavenge ABTS^+^ radicals is affected by several factors such as enzymes, DH, solubility of hydrolysates and MW of peptides. The ORAC-FL method evaluates the scavenging capacity due to a hydrogen-atom transfer mechanism. The antioxidant compound is exposed to a peroxyl radical generator (AAPH) and the oxidative degradation of fluorescein is measured (Ou et al. 2001). This assay uses a biological radical source, so is considered the most relevant method from a biological point of view, integrating the degree and time of antioxidant reaction (López-Pedrouso et al. 2020). The value of the peroxyl radical scavenging activity of porcine PH extracts was 87.7 ± 6.3 mg Trolox equivalent/g of dry extract. Indeed, porcine liver protein hydrolysates from enzymatic hydrolysis with several enzymes such as alcalase, bromelain, flavourzyme and papain have shown antioxidant capacity using ORAC-FL method (López-Pedrouso et al. 2020; Borrajo et al. 2020b). Our results corroborated this, proving that antioxidant peptides from porcine by-products can protect cells from oxidative damage.

Thus, these antioxidant peptides can be employed to maintain human health and also food safety and quality, by mitigating oxidative stress and lipid peroxidation triggered by free radicals produced during oxidation reactions of the human body and food products. Thereby, antioxidant peptides have received noteworthy attention in the food industry as functional ingredients and food additives. The use of synthetic antioxidants agents in the food industry is under strict regulation due to their side-effects on human health, namely induction of DNA damage and toxicity. Consequently, substituting synthetic antioxidants by natural antioxidants has become required (Tadesse and Emire, 2020).

### Inorganic fraction: CaP-based compounds

#### From inorganic residues to CaP: thermal treatment

To extract CaP phases from natural sources, a thermal treatment (calcination) is generally performed; this is done to remove possible organic fragments still present in the residues, as well as increasing the crystallinity of the obtained CaP (Piccirillo et al. 2014).

**Fig. 3:**
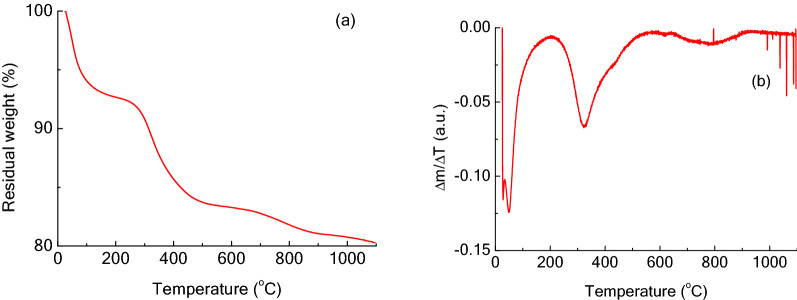
**a** Thermogravimetric analysis (TGA) of the inorganic residues; **b** first derivative of the curve

To understand the changes taking place during the heating and, therefore, to choose the best temperature for the calcination, a thermogravimetric analysis was carried out on the mineral residues; Fig. 3a shows the results, while Fig. 3b reports the first derivative of the curve, to better visualize the different steps.

The first weight loss (about 8%, *T* < 200 °C) is due to the removal of water, either adsorbed on the surface or included in the structure of the powder. A slightly larger loss (about 10%) is observed for 200 < *T* < 600 °C; losses in this temperature interval are associated with the burning of the residual organic fragments present in the material. This weight loss is much smaller to that previously observed for porcine bones—about 30% (Figueiredo et al. 2010); this difference confirming that a significant amount of organic matter was removed from the powder during the enzymatic hydrolysis. For higher temperatures, a further decrease in weight can be observed, although it is small (< 3%); this could be due to the removal of the carbonate present in the material (Figueiredo et al. 2010).

#### CaP characterization

Based on these results, it was decided to perform the calcination of the material at 700 °C; this value was chosen as the organic fragments were already removed but some carbonate ions were still present in the material—indeed, literature reports that the presence of such ions can be beneficial for bone-like cellular growth and bioactivity (Nakamura et al. 2016). Calcination at this temperature led to a yield of about 170 g, i.e., 17%.

Elemental analysis was performed to determine the content of calcium and phosphorus, as well as the Ca/P molar ratio—see Table 3.

**Table 3:** Calcium and phosphorus content (wt %) and Ca/P molar ratio for the non-calcined powder and CaP sample

Sample	Ca (%)	P(%)	Ca/P	Yield of the process (%)
Non-calcined powder	34.40 ± 0.28	15.25 ± 0.07	1.74 ± 0.01	20.5
CaP	40.30 ± 0.28	18.90 ± 0.42	1.65 ± 0.03	17.0

It can be seen that, although both calcium and phosphorus show higher relative content after the calcination, the increase is different for the two elements; in fact, the Ca/P ratio decreases slightly—from 1.74 to 1.65. This indicates that a small quantity of calcium is lost during the calcination; this behavior was previously observed for CaP derived from natural sources (Aydin et al. 2020). The Ca/P ratio for the CaP powder is very close to the stoichiometric one, that is 1.67.

**Fig. 4:**
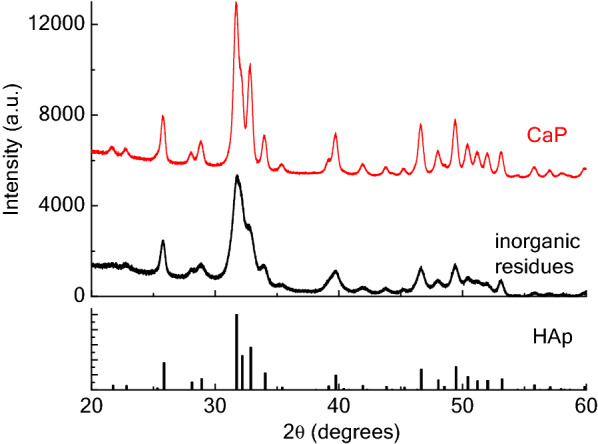
XRD data for the inorganic residue (non-calcined powder) and the CaP sample. The patterns are compared to the 01-072-1234 standard for HAp

Figure 4 shows the XRD patterns for the inorganic powder, prior the calcination and after (CaP). It can be seen that in both cases the only phase present is HAp; no other phosphate compounds, for instance β-tricalcium phosphate (β-TCP), are present. This was expected, the Ca/P ratio being not statistically different from the stoichiometric one; literature reports the formation of β-TCP for smaller Ca/P ratios, i.e., values close to 1.5 (Piccirillo et al. 2014). It can also be observed that CaP is much more crystalline than the starting non-calcined powder (sharper peaks).

**Fig. 5:**
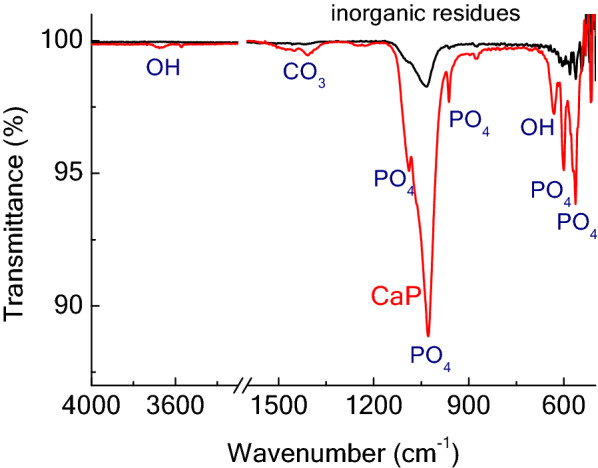
FTIR of the inorganic residues (i.e., non-calcined powder) and of the calcined CaP sample

FTIR spectra of the samples are shown in Fig. 5. It can be seen that the signals are much sharper and more resolved for the calcined CaP sample, due to its higher crystallinity. Peaks belonging to the HAp phosphate ions can be observed at 1090, 1040, 960, 603, 568 cm^−1^ (Piccirillo et al. 2013); it is interesting to note that no peak is present at 1122 cm^−1^. This signal corresponds to the β-TCP (Piccirillo et al. 2013); its absence confirms that this phase is not formed in the CaP sample, in agreement with XRD data (Fig. 4). Other weaker peaks present in the spectrum correspond to the OH ions at 3570 and 634 cm^−1^; moreover, signals at 1412 and 1450 cm^−1^ belong to the carbonate group (Figueiredo et al. 2010). These signals confirm that, after a treatment at 700 °C carbonate ions are still present in the HAp lattice.

**Fig. 6:**
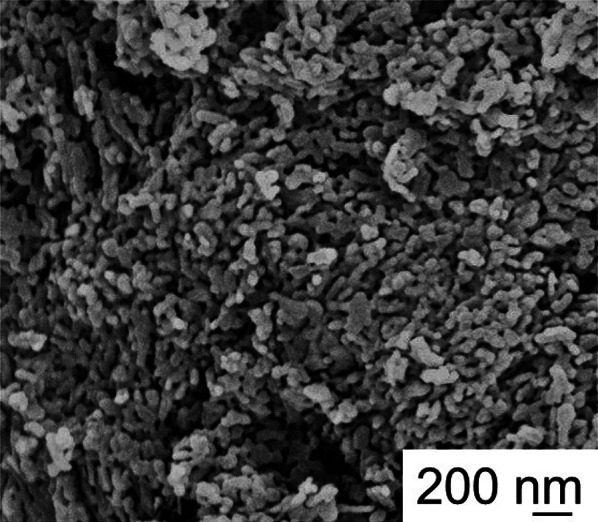
SEM micrograph of the calcined powder CaP

Figure 6 shows a SEM micrograph of the CaP sample. It can be seen that the powder has a nanometric structure; indeed particles with average size of about 50 nm can be observed. Literature data report that HAp from natural sources can be nanometric or not, depending on the sources (Barakat et al. 2009; Santana et al. 2019). The use of HAp in the form of nanoparticles has recently gained increasing attention, as it can show enhanced sintering properties, if compared to the micrometric powder (György et al. 2019), due to a higher specific surface and consequently a higher powder reactivity. Moreover, nano-HAp was also preferred for the preparation of composites with other compounds (i.e., biopolymers) (Turon et al. 2017). Compared with micrometric HAp, the nanoscale one induces better cellular functions like osteoblast responses (such as adhesion, proliferation, and differentiation) (Li et al. 2013). Also, for its application as a heavy metal remover, nano-HAp showed enhanced performance (Kowthaman and Varadappan 2019).

Based on these results, it can be stated that HAp with interesting properties and potential for application in biomedicine (for instance, as bone substitute) and as a heavy metal remover was successfully extracted in this combined process. The use of natural HAp has been explored instead to synthetic HAp, because it has comparable metabolic activity, preserves chemical composition and structure of the precursor material (Boutinguiza et al. 2012). In addition, there is a growing concern to develop clean, non-toxic and environmentally friendly procedures for HAp synthesis (with lower impact on the environment). It is required to reuse waste not only because waste materials are accumulating, but also because natural raw materials are being exhausted.

### Evaluation of the process

A full environmental and economic assessment of the process is beyond the scope of this work; some general comments, however, can already be made.

As reported in Sect. 3.1, the overall yield is about 30%; this value is well below the 100% associated with the full valorization. It is worth highlighting, however, that without performing this combined process, only PH or CaP would be extracted, with an overall minor yield and a fewer effective by-products valorization. Moreover, the residues also contain other organic fractions, such as lipids; these phases should also be considered to achieve a full valorization. The process presented here represent the first step to extract different parts from the residues; other step(s) for other fraction(s) could be added.

The extraction of PH was performed with an enzymatic bioprocess, without employing toxic solvents, according to the principles of the green chemistry, as an aqueous solution was used. For CaP, on the other hand, no solvent was necessary, but a simple thermal process was performed. Although energy is used for this treatment, with an associated impact on the environment, it has to be highlighted that the conventional routes for CaP production are likely to have a greater environmental impact. With these, in fact, chemical reactions between Ca and P are performed; in addition to the energy cost linked to this, the use of Ca- and P-containing reagents, derived from non-renewable sources, has to be considered. Both elements, in fact, are obtained from mining activities, whose impact is known to be quite high (Dubsok and Kittipongvieses 2016; Petrov and Danilov 2020). Considering this, by-product valorization surely offers a more sustainable solution.

From the economic point of view, both PH and CaP have high market value. Considering CaP, it is a good quality powder, with high purity and high level of crystallinity for biomedical applications, can cost up to tens of euros per gram. PH, for feed applications, is about 5x more valuable than a non-hydrolyzed protein. This makes the process profitable. This makes the process profitable.

## Conclusions

Porcine by-products (meat and bones) are a valuable source to produce natural value-added compounds for different markets. The combined process described in this work shows that it is possible to extract different compounds, both organics and minerals—indeed protein hydrolysates and hydroxyapatite were obtained. Overall, about 30% of the by-products were converted into valuable compounds—13.5% and 17% of protein hydrolysates and hydroxyapatite, respectively.

Protein hydrolysates were rich in low MW peptides and showed significant antioxidant properties. Hydroxyapatite, on the other hand, was shown to be single-phase and with a nanometric structure.

The proposed combined process is quite simple, cheap and easily scalable; all these features make it applicable at industrial scale, to achieve a more complete valorization of porcine by-products. Moreover, in principle the process could be applied also to by-products of other meat or fish industries. As future work, other steps could be added to the process, for the extraction of other phases (i.e., lipids) and to achieve a more complete valorization.

## Data Availability

All data supporting this article’s conclusion are available.

## Abbreviations

AAPH: 2,2’-Azobis(2-amidinopropane) dihydrochloride
ABTS: 2,2-Azino-bis-3-ethylbenzothiazoline-6-sulphonic acid
ATR: Attenuated total reflectance
CaP: Calcium phosphates
DH: Degree of hydrolysis
FPLC: Fast protein liquid chromatography
FTIR: Fourier transformed infrared spectroscopy
HAp: Hydroxyapatite
mAU: Milli absorbance units
MW: Molecular weight
ORAC-FL: Oxygen radical absorbance capacity
PH: Protein hydrolysates
RSA: Radical scavenging activity
SEM: Scanning electron microscopy
TCP: Tricalcium phosphate
TGA: Thermal analysis
TNBS: Trinitrobenzenesulfonic acid solution
XRD: X-ray diffraction

## References

[CR1] Ahmadifard N, Murueta JHC, Abedian-Kenari A, Motamedzadegan A, Jamali H (2016). Comparison the effect of three commercial enzymes for enzymatic hydrolysis of two substrates (rice bran protein concentrate and soy-been protein) with SDS-PAGE. J Food Sci Technol.

[CR2] Ahmed M, Verma AK, Patel R (2020). Collagen extraction and recent biological activities of collagen peptides derived from sea-food waste: a review. Sustain Chem Pharm.

[CR3] Ajibola CF, Fashakin JB, Fagbemi TN, Aluko RE (2011). Effect of peptide size on antioxidant properties of African yam bean seed (*Sphenostylis stenocarpa*) protein hydrolysate fractions. Int J Mol Sci.

[CR4] Albenzio M, Santillo A, Caroprese M, Della Malva A, Marino R (2017). Bioactive peptides in animal food products. Foods.

[CR5] Álvarez C, Rendueles M, Díaz M (2012). Production of porcine hemoglobin peptides at moderate temperature and medium pressure under a nitrogen stream. Functional and antioxidant properties. J Agric Food Chem.

[CR6] AOAC (1995) Official methods of analysis 16th Ed. Association of official analytical chemists. Washington DC, USA

[CR7] Aspevik T, Oterhals Å, Rønning SB, Altintzoglou T, Wubshet SG, Gildberg A, Afseth NK, Whitaker RD, Lindberg D (2017). Valorization of proteins from co-and by-products from the fish and meat industry. Chem Chem Technol Waste Valoriz.

[CR8] Aydin G, Terzioğlu P, Öğüt H, Kalemtas A (2020). Production, characterization, and cytotoxicity of calcium phosphate ceramics derived from the bone of meagre fish, Argyrosomus regius. J Austr Ceram Soc.

[CR9] Barakat NAM, Khil MS, Omran AM, Sheikh FA, Kim HY (2009). Extraction of pure natural hydroxyapatite from the bovine bones bio waste by three different methods. J Mater Process Technol.

[CR10] Borrajo P, López-Pedrouso M, Franco D, Pateiro M, Lorenzo JM (2020). Antioxidant and antimicrobial activity of porcine liver hydrolysates using flavourzyme. Appl Sci.

[CR11] Borrajo P, Pateiro M, Gagaoua M, Franco D, Zhang W, Lorenzo JM (2020). Evaluation of the antioxidant and antimicrobial activities of porcine liver protein hydrolysates obtained using alcalase, bromelain, and papain. Appl Sci.

[CR12] Boutinguiza M, Pou J, Comesaña R, Lusquiños F, De Carlos A, León B (2012). Biological hydroxyapatite obtained from fish bones. Mater Sci Eng, C.

[CR13] Chang C-Y, Wu K-C, Chiang S-H (2007). Antioxidant properties and protein compositions of porcine haemoglobin hydrolysates. Food Chem.

[CR14] Damgaard TD, Otte JAH, Meinert L, Jensen K, Lametsch R (2014). Antioxidant capacity of hydrolyzed porcine tissues. Food Sci Nutr.

[CR15] Damgaard T, Lametsch R, Otte J (2015). Antioxidant capacity of hydrolyzed animal by-products and relation to amino acid composition and peptide size distribution. J Food Sci Technol.

[CR16] de Queiroz ALM, Bezerra TKA, de Freitas PS, da Silva MEC, de Almeida GCA, Gadelha TS, Pacheco MTB, Madruga MS (2017). Functional protein hydrolysate from goat by-products: Optimization and characterization studies. Food Biosci.

[CR17] dos Santos Cardoso M, Godoy AC, Oxford JH, Rodrigues R, dos Santos CM, Bittencourt F, Signor A, Boscolo WR, Feiden A (2020). Apparent digestibility of protein hydrolysates from chicken and swine slaughter residues for Nile tilapia. Aquaculture.

[CR18] Dubsok A, Kittipongvieses S (2016). Estimated greenhouse gases emissions from mobile and stationary sources in the limestone and basalt rock mining in Thailand. Am J Environ Sci.

[CR19] Ferraro V, Gaillard-Martinie B, Sayd T, Chambon C, Anton M, Santé-Lhoutellier V (2017). Collagen type I from bovine bone. Effect of animal age, bone anatomy and drying methodology on extraction yield, self-assembly, thermal behaviour and electrokinetic potential. Int J Biol Macromol.

[CR20] Figueiredo M, Fernando A, Martins G, Freitas J, Judas F, Figueiredo H (2010). Effect of the calcination temperature on the composition and microstructure of hydroxyapatite derived from human and animal bone. Ceram Int.

[CR21] Fu Y, Liu J, Hansen ET, Bredie WLP, Lametsch R (2018). Structural characteristics of low bitter and high umami protein hydrolysates prepared from bovine muscle and porcine plasma. Food Chem.

[CR22] Fu Y, Bak KH, Liu J, De Gobba C, Tøstesen M, Hansen ET, Petersen MA, Ruiz-Carrascal J, Bredie WLP, Lametsch R (2019). Protein hydrolysates of porcine hemoglobin and blood: Peptide characteristics in relation to taste attributes and formation of volatile compounds. Food Res Int.

[CR23] Gharibzahedi SMT, Jafari SM (2017). The importance of minerals in human nutrition: Bioavailability, food fortification, processing effects and nanoencapsulation. Trends Food Sci Technol.

[CR24] György S, Károly Z, Fazekas P, Németh P, Bódis E, Menyhárd A, Kótai L, Klébert S (2019). Effect of the reaction temperature on the morphology of nanosized HAp. J Therm Anal Calorim.

[CR25] Han K-H, Shimada K, Hayakawa T, Yoon TJ, Fukushima M (2014). Porcine Splenic Hydrolysate has Antioxidant Activity in vivo and in vitro. Korean J Food Sci Anim Resour.

[CR26] Irshad I, Kanekanian A, Peters A, Masud T (2015). Antioxidant activity of bioactive peptides derived from bovine casein hydrolysate fractions. J Food Sci Technol.

[CR27] Khan HM, Iqbal T, Ali CH, Yasin S, Jamil F (2020). Waste quail beaks as renewable source for synthesizing novel catalysts for biodiesel production. Renew Energy.

[CR28] Kowthaman CN, Varadappan AMS (2019). Synthesis, characterization, and optimization of Schizochytrium biodiesel production using Na+-doped nanohydroxyapatite. Int J Energy Res.

[CR29] Lapeña D, Vuoristo KS, Kosa G, Horn SJ, Eijsink VGH (2018). Comparative assessment of enzymatic hydrolysis for valorization of different protein-rich industrial byproducts. J Agric Food Chem.

[CR30] Li B, Chen F, Wang X, Ji B, Wu Y (2007). Isolation and identification of antioxidative peptides from porcine collagen hydrolysate by consecutive chromatography and electrospray ionization–mass spectrometry. Food Chem.

[CR31] Li X, Wang L, Fan Y, Feng Q, Cui FZ, Watari F (2013). Nanostructured scaffolds for bone tissue engineering. J Biomed Mater Res, Part A.

[CR32] Li T, Shi C, Zhou C, Sun X, Ang Y, Dong X, Huang M, and Zhou G (2020) Purification and characterization of novel antioxidant peptides from duck breast protein hydrolysates. LWT: 109215.

[CR33] Liu Q, Kong B, Xiong YL, Xia X (2010). Antioxidant activity and functional properties of porcine plasma protein hydrolysate as influenced by the degree of hydrolysis. Food Chem.

[CR34] López-Pedrouso M, Borrajo P, Pateiro M, Lorenzo JM, Franco D (2020). Antioxidant activity and peptidomic analysis of porcine liver hydrolysates using alcalase, bromelain, flavourzyme and papain enzymes. Food Res Int.

[CR35] Lü X Y, Fan Y B, Gu D, and Cui W(2007) Preparation and characterization of natural hydroxyapatite from animal hard tissues. In, 213–16. Trans Tech Publ

[CR36] Meshginfar N, Sadeghi-Mahoonak A, Ziaiifar AM, Ghorbani M, Kashaninejad M (2014). Study of antioxidant activity of sheep visceral protein hydrolysate: Optimization using response surface methodology. ARYA Atherosclerosis.

[CR37] Nakamura M, Hiratai R, Hentunen T, Salonen J, Yamashita K (2016). Hydroxyapatite with high carbonate substitutions promotes osteoclast resorption through osteocyte-like cells. ACS Biomater Sci Eng.

[CR38] Nie Y, Hou Q, Bai C, Qian H, Bai X, Ju M (2020). Transformation of carbohydrates to 5-hydroxymethylfurfural with high efficiency by tandem catalysis. J Clean Prod.

[CR39] Ou B, Hampsch-Woodill M, Prior RL (2001). Development and validation of an improved oxygen radical absorbance capacity assay using fluorescein as the fluorescent probe. J Agric Food Chem.

[CR40] Petrov DS, Danilov AS (2020). Analysis and assessment of the hydrochemical conditions of flooded phosphate rock quarries. Water Ecol.

[CR41] Piccirillo C, Silva MF, Pullar RC, Da Cruz IB, Jorge R, Pintado MME, Castro PML (2013). Extraction and characterisation of apatite-and tricalcium phosphate-based materials from cod fish bones. Mater Sci Eng, C.

[CR42] Piccirillo C, Pullar RC, Tobaldi DM, Castro PML, Pintado MME (2014). Hydroxyapatite and chloroapatite derived from sardine by-products. Ceram Int.

[CR43] Re R, Pellegrini N, Proteggente A, Pannala A, Yang M, Rice-Evans C (1999). Antioxidant activity applying an improved ABTS radical cation decolorization assay. Free Radical Biol Med.

[CR44] Safavi A, Mohammadi A, Sorouri M (2020). Cobalt-nickel wrapped hydroxyapatite carbon nanotubes as a new catalyst in oxygen evolution reaction in alkaline media. Electrocatalysis.

[CR45] Saiga AI, Tanabe S, Nishimura T (2003). Antioxidant activity of peptides obtained from porcine myofibrillar proteins by protease treatment. J Agric Food Chem.

[CR46] Santana CA, Piccirillo C, Pereira SIA, Pullar RC, Lima SM, Castro PML (2019). Employment of phosphate solubilising bacteria on fish scales–Turning food waste into an available phosphorus source. J Environ Chem Eng.

[CR47] Santos AF, Arim AL, Lopes DV, Gando-Ferreira LM, Quina MJ (2019). Recovery of phosphate from aqueous solutions using calcined eggshell as an eco-friendly adsorbent. J Environ Manage.

[CR48] Sousa P, Borges S, Pintado M (2020). Enzymatic hydrolysis of insect Alphitobius diaperinus towards the development of bioactive peptide hydrolysates. Food Funct.

[CR49] Tacias-Pascacio V G, Morellon-Sterling R, Siar E-H, Tavano O, Berenguer-Murcia Á, and Fernandez-Lafuente R (2020) Use of Alcalase in the production of bioactive peptides: A review. Int J Biol Macromol 10.1016/j.ijbiomac.2020.10.060 33091472

[CR50] Tadesse SA, Emire SA (2020). Production and processing of antioxidant bioactive peptides: a driving force for the functional food market. Heliyon.

[CR51] Toldrá F, Mora L, Reig M (2016). New insights into meat by-product utilization. Meat Sci.

[CR52] Turon P, Del Valle LJ, Alemán C, Puiggalí J (2017). Biodegradable and biocompatible systems based on hydroxyapatite nanoparticles. Appl Sci.

[CR53] Verma AK, Chatli MK, Kumar P, Mehta N (2017). Antioxidant and antimicrobial activity of protein hydrolysate extracted from porcine liver. Indian J Anim Sci.

[CR54] Wang JZ, Zhang HAO, Zhang M, Yao WT, Mao XY, Ren FZ (2008). Antioxidant activity of hydrolysates and peptide fractions of porcine plasma albumin and globulin. J Food Biochem.

[CR55] Zou Z, Wei M, Fang J, Dai W, Sun T, Liu Q, Gong G, Liu Y, Song S, Ma F (2019). Preparation of chondroitin sulfates with different molecular weights from bovine nasal cartilage and their antioxidant activities. Int J Biol Macromol.

